# Observations on an Epidemic Scarlet Fever and Sore Throat; Communicated to Dr. Bradley

**Published:** 1803-02-01

**Authors:** R. Freeman

**Affiliations:** Cheltenham in Gloucestershire


*Observations on an* Epidemic Scarlet Fever *and* Sore Throat; *communicated to Dr. Bradley by* Mr. R. Freeman, *of* Cheltenham *in Gloucestershire.*

I HAVE been induced to draw up the following account of the Scarlet Fever and Sore Throat, which has for some time been an unwelcome sojourner in our neighbourhood, in consequence of a general request made by the Editors of the Medical Journal, in the last number of that publication, for Communications respecting the prevailing epidemics of the present season. In submitting it to your inspection, I am aware it will not be in my power to advance vance any thing new or important to the science of medicine; so much has already been said on the subject of scarlatina by authors of the first celebrity, that it may be presumed little remains for a practitioner of the present day but to repeat their descriptions and enforce their methods of cure; yet may I be allowed to say, there seems a want of due discrimination in most of our nosologists and other writers when treating of these diseases, in so much, that was it not for their prevalence as an epidemic, young practitioners might frequently be deceived in forming their diagnosis. Cullen gives us two varieties, viz. the scarlatina simplex, and the scarlatina cynanchia. Sauvage furnishes us with an outline of each species, distinguishing the latter by the very appropriate term anginosa. Vogel and Sagar describe the milder disease only. Macbride names the two varieties, scarlatina benigna and scarlatina maligna. For the former of these varieties we are referred to the authority of Sydenham, who describes it as a mild disease, without danger, requiring only a regulated diet and confinement, and unattended with any affection of the throat. Morton, in his book on inflammatory fevers, describes the symptoms of both these species of scarlatina with his accustomed accuracy, but at the same time seems desirous to confound the disease altogether with the measles, from which he asserts it to differ only in the appearance of the eruption; other writers have adopted the same indiscriminating idea. In the course of forty years practice, we are told by Cullen, he has never once met with the milder species of the disease, but in every instance where he has seen it prevail, it has uniformly been of the latter variety; which he proceeds to desciibe in terms of much ambiguity, expressing doubts whether it might not, in many instances, be mistaken for the cynanche maligna. Dr. Withering has given the world a very clear and satisfactory account of its appearance at Birmingham in the year 1778, in which instance it seems to have assumed its most malign aspect; he allows, that in some instances it bears a close resemblance to the ulcerated sore throat, or cynanche maligna. As it may be deemed impertinent to continue this examination further, I shall here drop it by observing, that the disease in question is sufficient to furnish us with a proof of the imperfections of our best systematic arrangements, of the confusion and false confidence to which they may give rise, and of the many errors in practice which must necessarily ensue.

I will

I will proceed now to give some account of the scarlet fever, as it appeared in our neighbourhood: Its commencement may be fixed about the latter end of July; we are arrived at the middle of December, and have not yet witnessed its termination, though in consequence of the number of persons who have gone through the disease, it has for this month past been gradually on the decline. By far the greater number affected have been children, from infancy to the age of fifteen years; from that period to thirty years, include almost the whole of the remainder, and they have been, comparatively speaking, not many; of persons further advanced in life, very few have come under my observation. As it would lead me too far, was I to particularise every symptom, I shall content myself with pointing out the most prominent, and those which seem to constitute varieties in the disease. In general, the usual phenomena of inflammatory fever have, in the first instance, been accompanied with enlargements and indurations of the submaxillary glands, with a stiffness and rigidity of the muscles of the neck, and with a painful and difficult deglutition; upon inspecting the mouth, the uvula and fauces appear tumefied, and bear evident marks of inflammation, giving out an abundant secretion of mucus; the patient complains of head-ach, sickness, nausea, and total loathing of food; the alvine evacuations are most commonly of the usual quantity, the urine high coloured, and depositing a copious sediment; the pulse is weak, and varying from 110 to 130; the period at which the scarlet eruption makes its appearance varies also considerably; in some instances, it is seen on the first day of the fever, in others it is delayed to the second or third day; this eruption does not appear, as it is generally described in authors, in large blotches, but in small minute spots or streaks, occupying the whole surface of the body. In general, the third, fourth, and fifth days are the worst; during this time the heat of the skin is very great, the patient becomes languid, watchful, and frequently betrays a confusion of intellect; from the fifth day to the eighth the symptoms gradually wear off, leaving the person in a state of debility, which, however, does not prove of long continuance; the scarlet colour disappears, in its stead the skin becomes brown, dry, and covered with minute branny scales, dropping off by friction; in some instances the cuticle entirely separates itself from the hands and feet. Such is the disease in its mildest and most general aspect. Three varieties varieties are distinguishable. 2. Where the tonsils, uvula and soft palate have been affected with sloughs and ulcerations, requiring the liberal use of detergent gargles; in some instances, the tongue has partook of the inflammation, when the appearance has much resembled aphthæ; this variety has produced nearly as many instances as the foregoing; the patients have been longer in recovering their health, and in some instances a putrid tendency has indicated the limited use of wine and antiseptic remedies. 3. A total absence of the scarlet eruption; and of this I have seen several instances; (indeed I was myself an instance, having contracted the disease in the month of September) every other characteristic symptom was strongly marked. 4. When the disease has from the commencement assumed a direct putrid tendency, differing in no respect from the cynanche maligna; every fatal case that has come to my knowledge may be referred to this variety; fortunately, it has produced but few instances.

In the treatment of such a disease as this, it is obvious we must be guided by the particular symptoms of each case, as it would be impossible to lay down an invariable line of conduct. Vomits, in the first instance, have been commended; and as far as my experience goes, with great propriety; their use, I think, should be confined to the commencement of the disease; in its advanced state they proved injurious; nor would I give them indiscriminately, but only in those cases where the sickness and nausea greatly prevail. Alkaline remedies have been pointed out as specifics in this disease; I cannot say I have found much advantage from their use. Bark, unless in cases where the septic tendency is very urgent, is certainly inadmissible, but it may be used with more propriety in the subsequent state of debility. Opium during the inflammatory state, whether in small or large doses, evidently did harm. Bleeding, either generally or topically, I never had recourse to. Though Dr. Withering has given us a strong caution toavoid the use of blisters, I have applied them to the chest in cases where the respiration has been difficult and oppressed with much advantage; they have also proved useful when applied to the enlarged glands of the neck; though I have more commonly used as a substitute an epithem with the aq. ammon. pur. which, by gently stimulating the skin, has afforded considerable relief. In fine, the experience of one epidemic proves, that the scarlatina cynanchia may in the generality of cases be very safely trusted to Nature, and and a mild antiphlogistic regimen; where they vary from the general and require the assistance of art, the symptoms are sufficiently defined to indicate to the practitioner the proper remedies he should apply.

*December* 20, 1802.

P. S. A late writer on Scarlatina, (Dr. Peart) takes upon himself considerable merit for propagating what he calls a discovery of his own, viz. that the ammonia preparata is a specific in this disease. To prove that this is no modern discovery (at least that its use is not such) it will be sufficient to quote a passage from Dr. Withering's Treatise; after commending the use of fixed alkali, he observes, "the volatile alkali may likewise be given with advantage, but it is difficult to get a sufficient quantity of it swallowed."

